# Supplementary material for: Septin organization is regulated by the Gpa1 ubiquitination domain and endocytic machinery during the yeast pheromone response
Source: Mol Biol Cell. 2025 Sep 12;36(10):ar125. doi: 10.1091/mbc.E25-05-0234 (PMC12483322; doi:10.1091/mbc.E25-05-0234)
Supplement: Supplementary file 1 [file mbc-36-ar125-s001.pdf]

Supplemental Materials

Molecular Biology of the Cell

Johnson *et al*.

Supplemental Figure S1

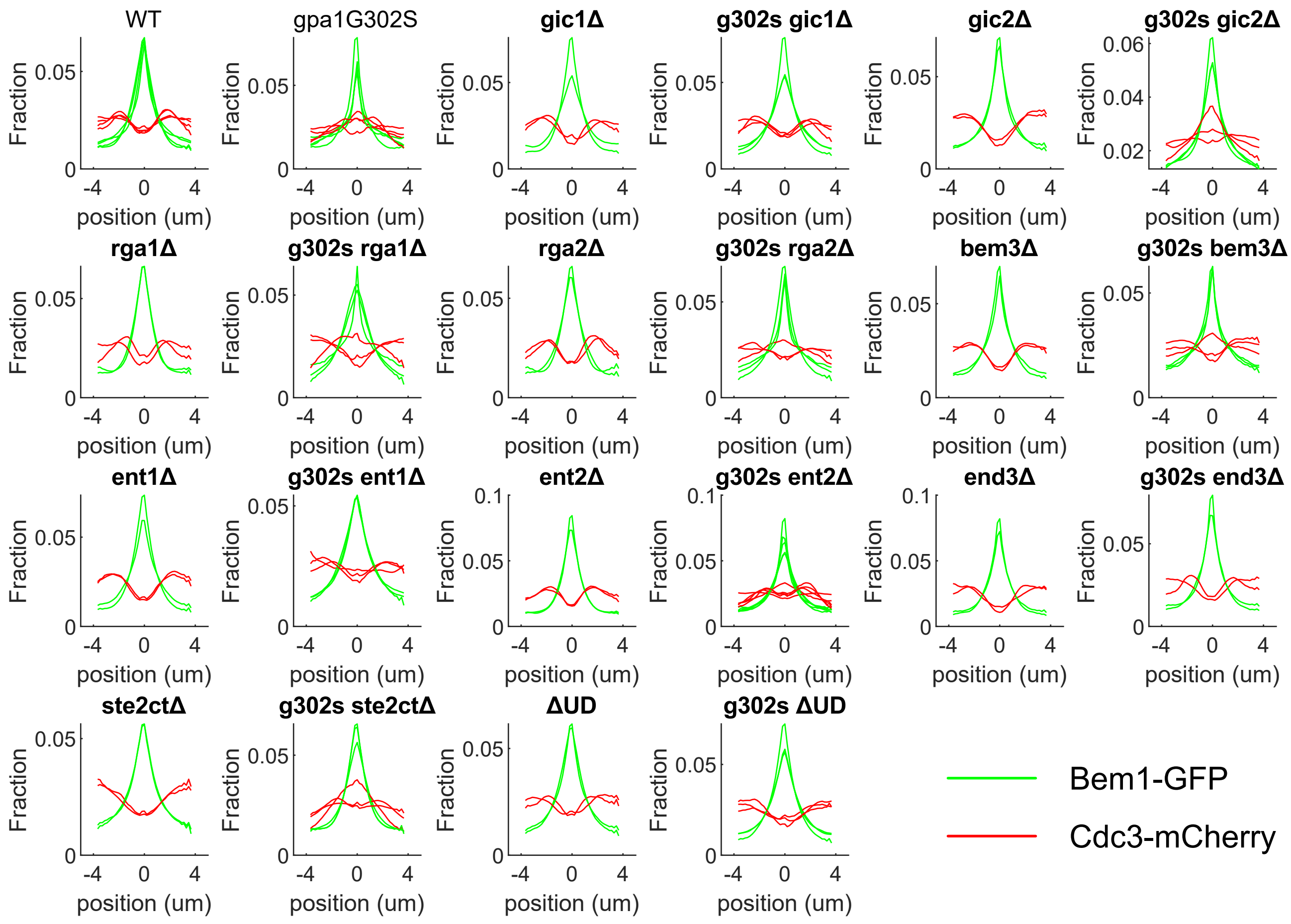

Supplemental Figure S1. Experiment-to-experiment Variability. The average Bem1 (green) and Cdc3 (red) profiles for each independent experiment for the indicated strain are shown. Strains with *gpa1^{G302S}* backgrounds show higher experiment-to-experiment variability.

Supplemental Figure S2

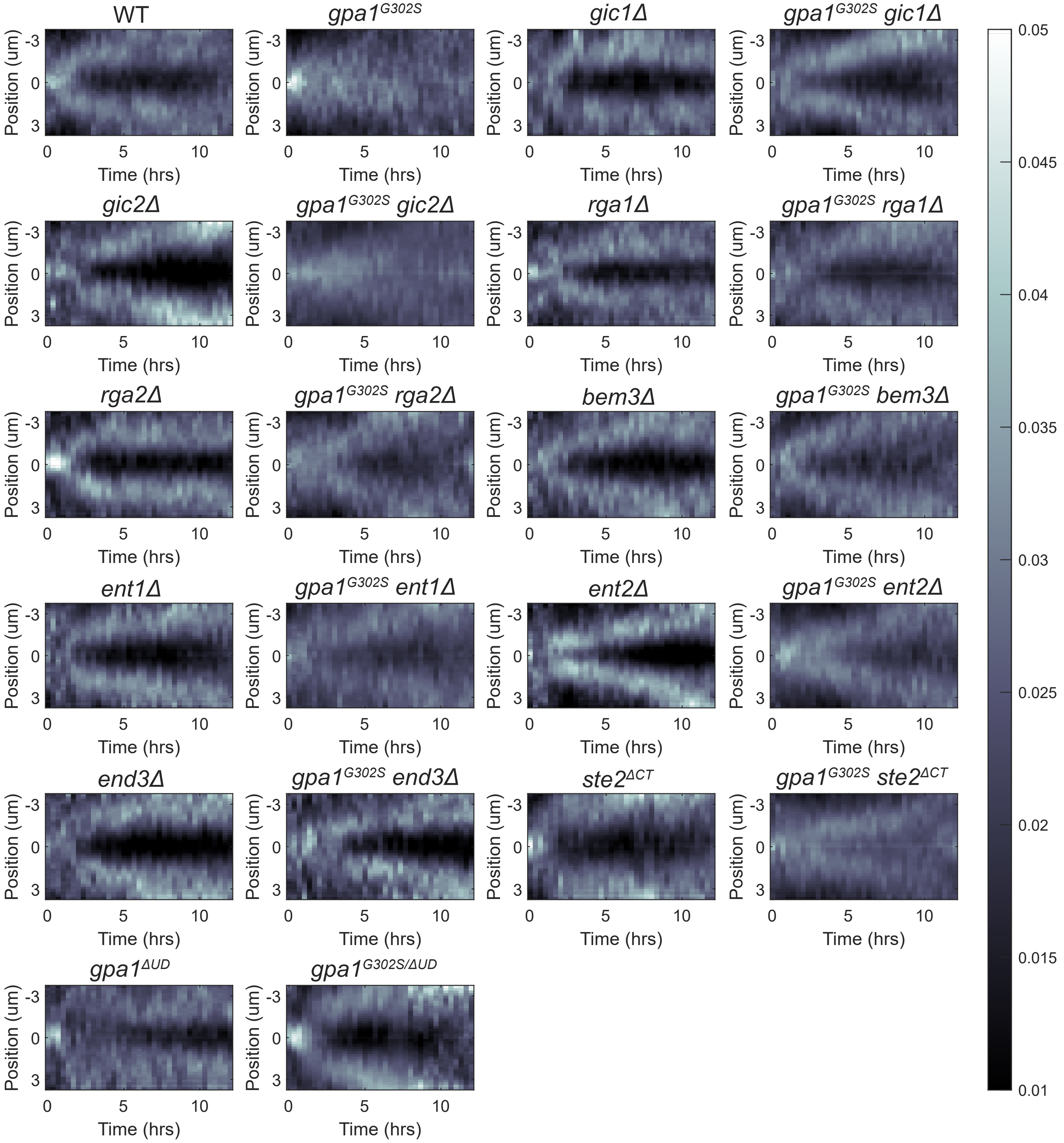

Supplemental Figure S2. Average Kymographs of Cdc3-mCherry. The average kymograph of Cdc3-mCherry spatially normalized to peak Bem1-GFP signal for every cell from the indicated strain.

Supplemental Figure S3

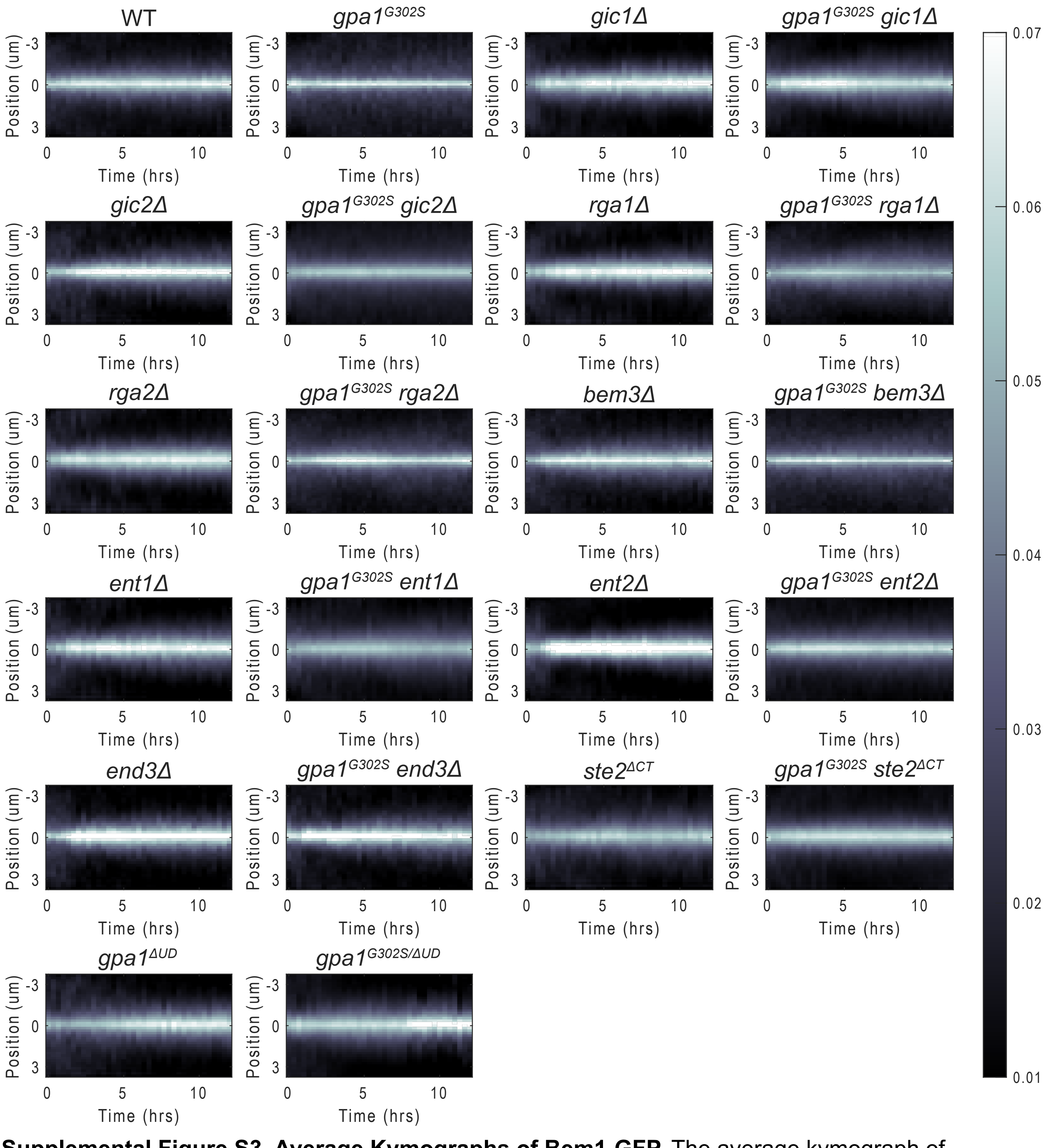

Supplemental Figure S3. Average Kymographs of Bem1-GFP. The average kymograph of Bem1-GFP spatially normalized to peak Bem1-GFP signal for every cell from the indicated strain.

Supplemental Figure S4

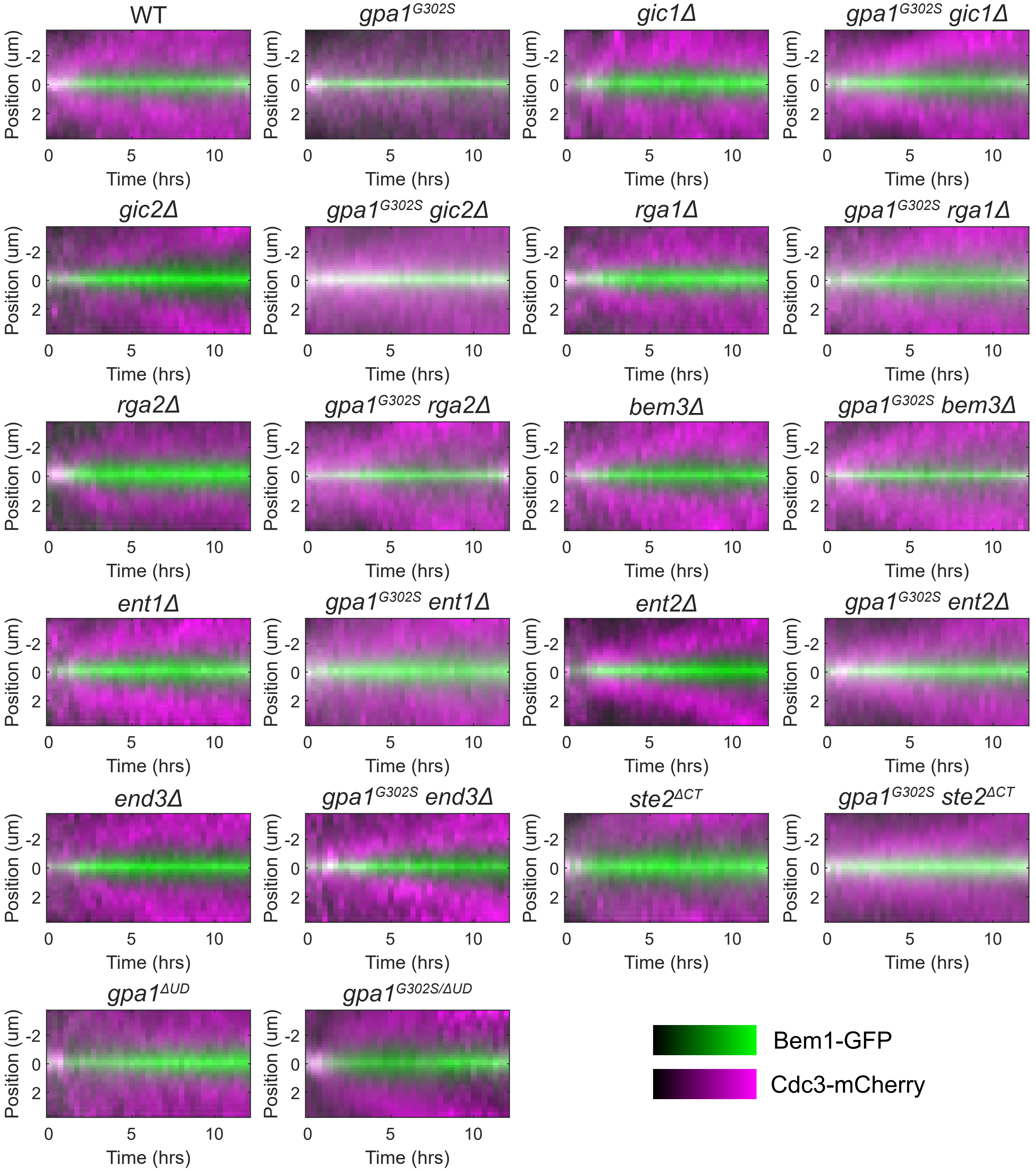

Supplemental Figure S4. Merged Average Kymographs. Merged images of the average kymographs of Cdc3-mCherry (magenta) and Bem1-GFP (green) from Supplemental Figures S2 and S3.

Supplemental Figure S5

GPA1 Cdc3-mCherry Kymographs from Individual Cells

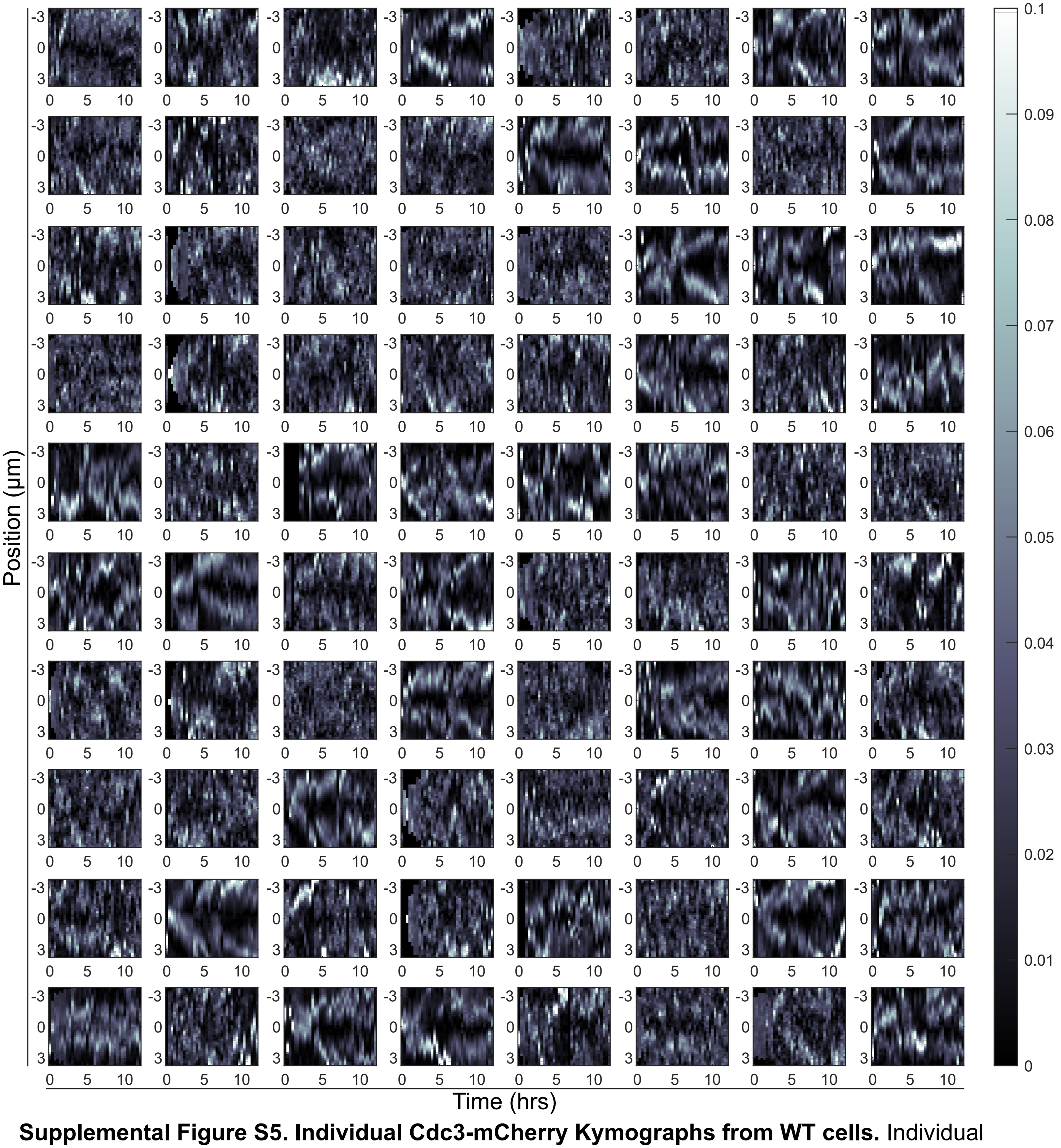

Supplemental Figure S5. Individual Cdc3-mCherry Kymographs from WT cells. Individual kymographs of Cdc3-mCherry from 80 individual WT GPA1 cells.

Supplemental Figure S6

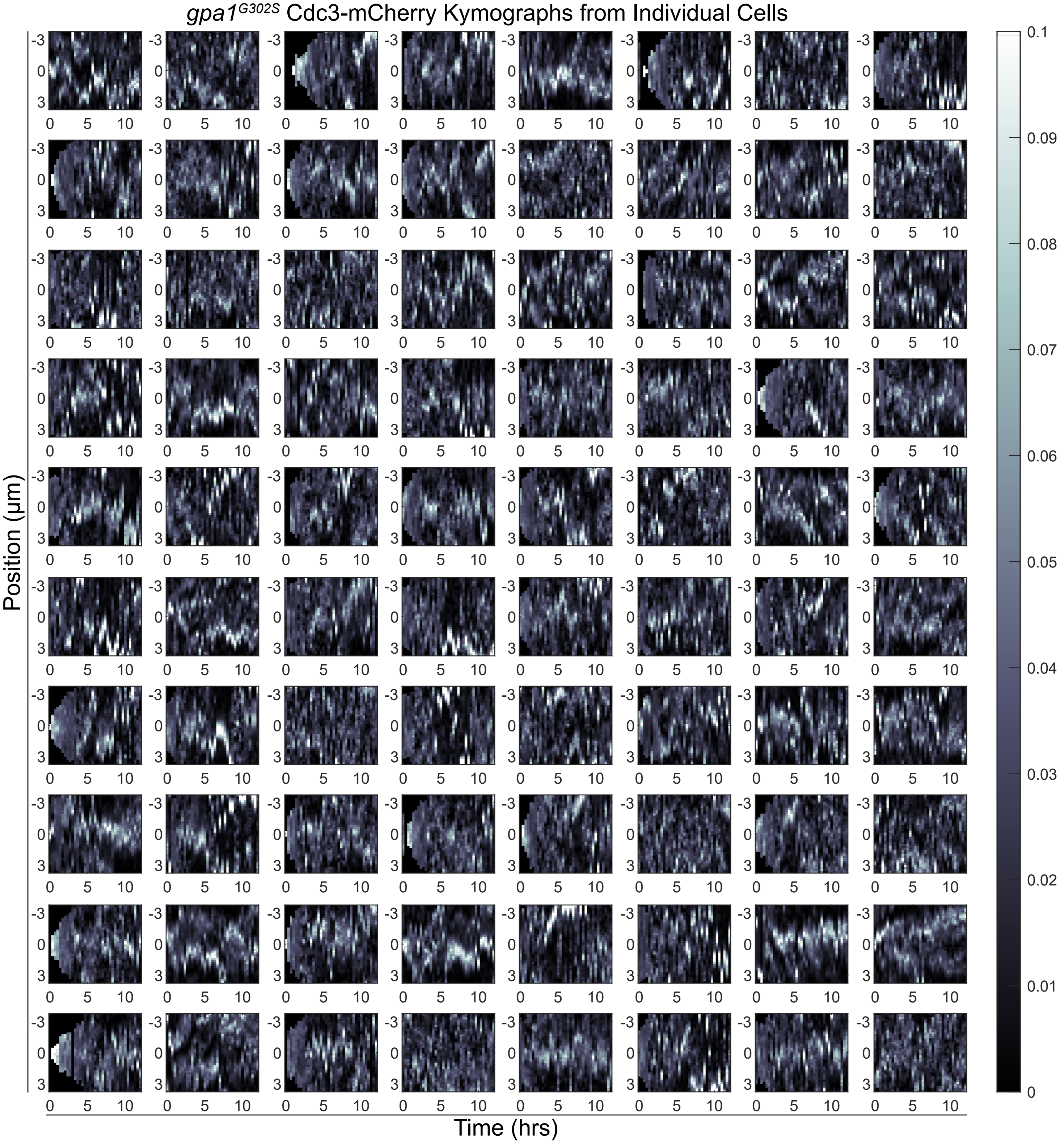

Supplemental Figure S6. Individual Cdc3-mCherry Kymographs from gpa1^{G302S} cells. Individual kymographs of Cdc3-mCherry from 80 individual *gpa1*^{G302S} cells.

Supplemental Video S7. Cells expressing *gpa1*$^{G302S\text{-}\Delta UD}$ require more pheromone to continue the mating response. Shown are DIC images of *gpa1*$^{G302S\text{-}\Delta UD}$ *Bem1-GFP Cdc3-mCherry* cells growing in a microfluidic device at either 300 nM or 1000 nM pheromone. Images are taken every 20 minutes for 12 hours.

Supplemental Figure S8

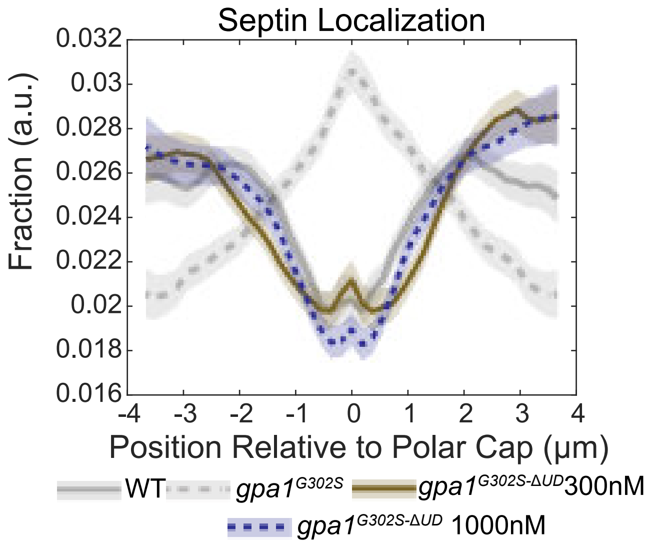

Supplemental Figure S8. Altered sensitivity to pheromone in gpa1ΔUD cells does not impact quantitation of septin organization. Quantitation of septin organization in cells expressing *gpa1*$^{G302S\text{-}\Delta UD}$ at 300 nM pheromone, conditions where they escape pheromone-induced arrest in G1, and at 1 μM pheromone, where cells stay arrested for the entire duration of the experiment.

Supplemental Figure S9

Sensitivity Analysis by Halo Assay

A)

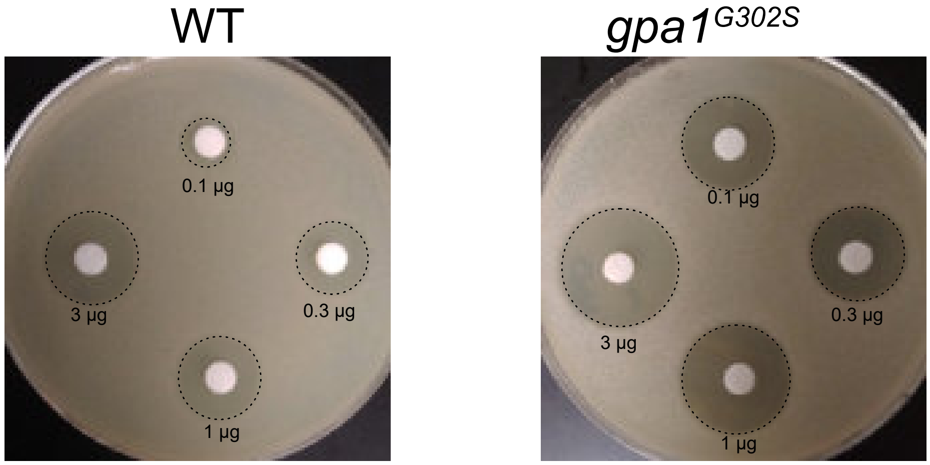

B)

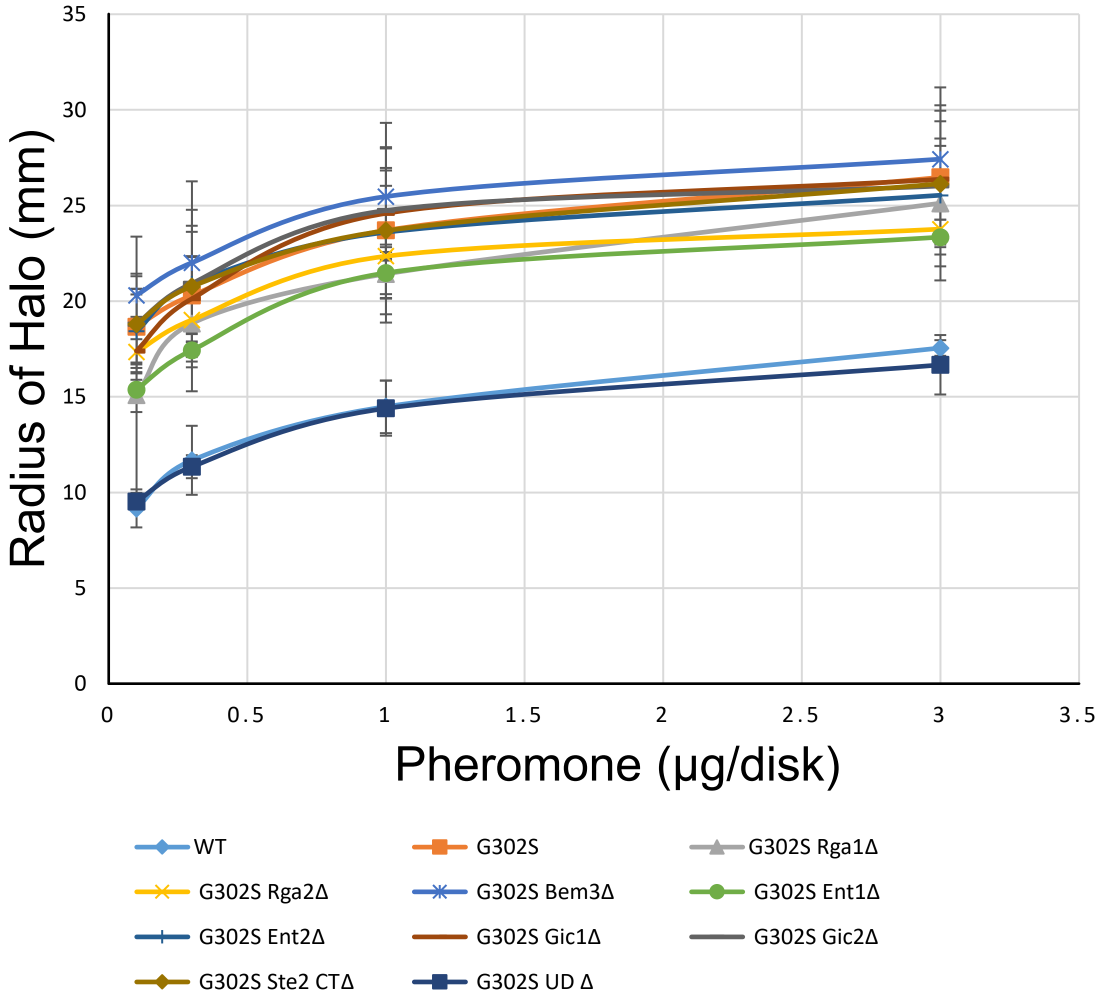

Supplemental Figure S9. Sensitivity of *gpa1^{G302S}* background strains. A) Halo assays were performed to assess the sensitivity of each strain to pheromone. Shown are examples of Halo Assays for WT and *gpa1^{G302S}*. Disks are labeled with the amount of pheromone present. B) Halo assays were quantified by measuring the halo radius (zone of no growth) around the indicated pheromone dose. Results were plotted as radius vs pheromone amount. Larger radii indicate higher sensitivity to pheromone. Each point is the average of 3 experiments. Error bars represent standard deviation.
